# Waterborne Polyurethane Dispersions and Thin Films: Biodegradation and Antimicrobial Behaviors

**DOI:** 10.3390/molecules26040961

**Published:** 2021-02-11

**Authors:** Samy A. Madbouly

**Affiliations:** 1 School of Engineering, Behrend College, Pennsylvania State University, Erie, PA 16563, USA; sum1541@psu.edu; Tel.: +814-595-7169; 2 Department of Chemistry, Faculty of Science, Cairo University, Giza 12613, Egypt

**Keywords:** waterborne polyurethane dispersions, biodegradable, antimicrobial, particle size, cationic polymerization, biomedical applications

## Abstract

Biodegradable and antimicrobial waterborne polyurethane dispersions (PUDs) and their casted solid films have recently emerged as important alternatives to their solvent-based and non-biodegradable counterparts for various applications due to their versatility, health, and environmental friendliness. The nanoscale morphology of the PUDs, dispersion stability, and the thermomechanical properties of the solid films obtained from the solvent cast process are strongly dependent on several important parameters, such as the preparation method, polyols, diisocyanates, solid content, chain extension, and temperature. The biodegradability, biocompatibility, antimicrobial properties and biomedical applications can be tailored based on the nature of the polyols, polarity, as well as structure and concentration of the internal surfactants (anionic or cationic). This review article provides an important quantitative experimental basis and structure evolution for the development and synthesis of biodegradable waterborne PUDs and their solid films, with prescribed macromolecular properties and new functions, with the aim of understanding the relationships between polymer structure, properties, and performance. The review article will also summarize the important variables that control the thermomechanical properties and biodegradation kinetics, as well as antimicrobial and biocompatibility behaviors of aqueous PUDs and their films, for certain industrial and biomedical applications.

## 1. Introduction

The solventborne polyurethane dispersions (PUDs) commonly used in the manufacturing of coatings, paints, and inks cause serious air quality problems due to the evaporation of large amounts of volatile organic compounds (VOCs) into the atmosphere during the formulation and application processes. The Local Air Quality Regulators of United States and the Environmental Protection Agency (EPA) have encouraged decreasing or eliminating the amounts of VOCs released to the atmosphere. Relatively new environmentally-friendly PUDs with almost no negative impact on the atmosphere have been developed in the last few years [1,2,3,4,5,6,7,8,9,10,11,12,13,14,15]. One convenient way to eliminate the VOCs in the manufacturing of PUDs in different industrial products, such as coatings, paints, and inks, is by replacing the expensive and harmful organic solvents with cost effective, environmentally benign solvents, such as water [1,2,3,4,5,6,7,8,9,10,11,12,13,14,15]. The cost effective and environmental-friendly aqueous PUDs are excellent alternatives to their solventborne counterparts and have the potential to greatly reduce environmental pollution compared to using costly and harmful organic solvents. A huge number of investigations aimed at developing and improving the manufacturing process and quality of waterborne PUDs have been published [16,17,18,19,20,21,22,23,24,25]. The early work of Dieterich and his co-workers in Bayer AG is very crucial because it pioneered the fundamental research in this area [16,17]. Recently, waterborne PUDs have been selected to replace the solventborne PUDs in various applications, such as coatings for various fibers, paint additives, defoamers, pigment pastes, and textile dyes, associate thickeners, adhesives for alternative substrates, primers for metals, caulking materials, and emulsion polymerization media for different monomers [18,19,20,21,22,23,24,25]. These environmentally friendly waterborne PUDs are expected to exhibit similar or superior performance compared to that of conventional solvent borne systems. In addition, aqueous PUDs are now one of the most rapidly developing and active branches of PU chemistry and technology as a result of their versatility and environmental friendliness. The PUDs are blends of different chemical species uniformly dispersed in nanoscale colloidal particles in a liquid continues phase. The properties of PUDs are strongly related to the interfacial forces and physicochemical interactions between the different chemical species in the dispersion. The colloidal dispersion can form stable dispersed particles (discontinuous phase) only if the particles are dispersed uniformly in the liquid medium (continuous phase). Unstable dispersions can be formed when the colloidal particles diffuse together, or coalescence to form a single bigger droplet that leads to a reduction in the total surface area. An unstable dispersion is also obtained when the dispersed colloidal particles agglomerate without a new particle (i.e., flocculation) [26,27,28,29,30,31]. For aqueous PUDs, the nanoscale polyurethanes are dispersed in water. Therefore, both the colloidal and chemical aspects of the dispersion, as well as the nature of the polyols, diisocyanates, internal surfactants, and neutralization degree will strongly affect the final properties of the film.

Generally, polyurethanes have unique chemical structures of microphase-separated alternating soft and hard segments. Polyurethane ionomers can be obtained by introducing ions into either hard or soft segments to yield a wide range of polyurethanes with prescribed chemical and mechanical properties [32,33]. In recent decades, extensive work has been completed for the synthesis of various kinds of polyurethanes and polyurethane ionomers with different properties and industrial applications [26,27,28,29,30,31,32,33,34,35,36]. Polyurethane (PU) ionomer dispersions can be synthesized by a reaction of diisocyanates, polyols, and dimethylol propionic acid (DMPA, internal surfactant) and a subsequent chain extension process [37,38,39]. 

**Figure 1:**
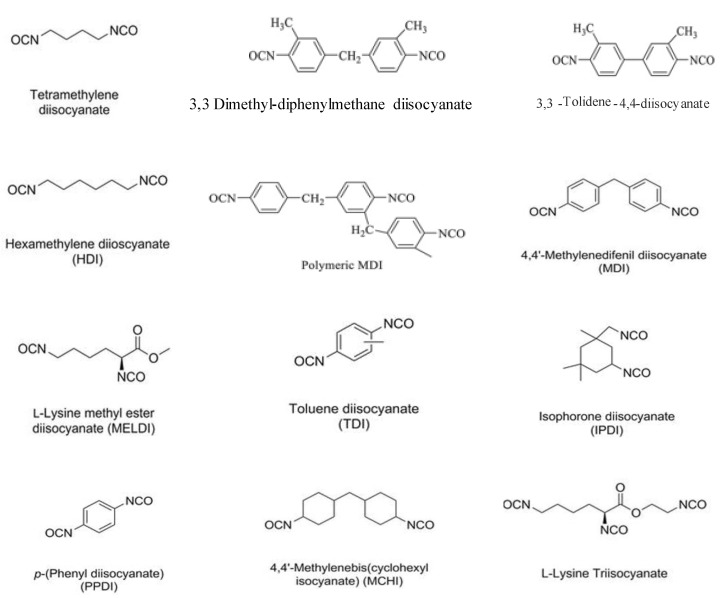
Diisocyanates commonly used for polyurethane synthesis (adapted with permission from [40]).

**Figure 2:**
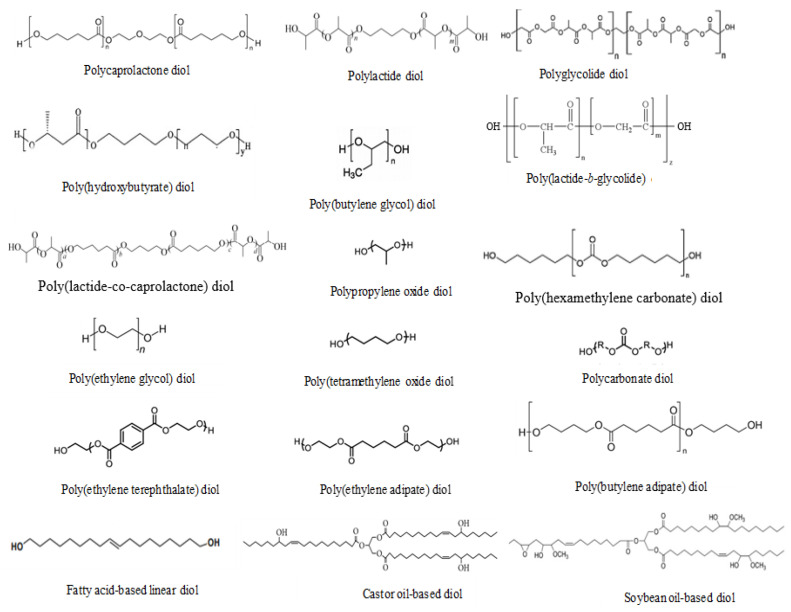
List of diols materials commonly used in polyurethane synthesis.

Polyurethane ionomers contain polar urethane groups that are capable of interacting via hydrogen bonds. The polyurethane ionomers have ionic groups that can aggregate and interact with each other, and attach to the hydrophobic neighborhood. With changing the molar mass of the segments, chemical structure, internal surfactants, degree of neutralization, and ratio of the soft to the hard segments, a broad range of physical and mechanical properties can be obtained. The materials can be soft and tacky, hard and brittle, or anywhere in between. Figure 1 depicts a list of different types of diisocyanates that are commonly used in the synthesis of polyurethanes for different industrial applications [40]. Aromatic and aliphatic diisocyanates have different chemical reactivities and can be used to tailor the mechanical, thermal and UV stability of polyurethanes. The aromatic diisocyanates are more reactive than the aliphatic ones and can be used in polyurethane synthesis only if their reactivities are matched with the specific polymer reaction to create desired properties in the final products. For instance, polyurethane coatings made from aromatic diisocyanates will undergo photodegradation [41], while coatings made from aliphatic isocyanates are light stable [42,43,44,45]. The soft segments of PU are commonly low molar mass (*M_w_* = 500–5000 g/mol) dihydroxy terminated long chain macroglycols (polyols), such as polyesters, polyethers, polydienes, polyolefins, and plant oil-based polyols (see Figure 2). PU made from polyester soft segments are very sensitive to hydrolytic degradation via cleavage of the ester linkage compared to polyether-based urethanes. Polyester polyols, with alkyl side groups, such as poly(2,4-diethyl-1,5-pentamethylene adipate) glycol (PDPAd) have been developed to improve the hydrolytic stability of waterborne PUDs [46]. Polyurethanes with a polyethylene oxide (PEO) soft segment have poor water resistance and exhibited a significant level of hydrolytic degradation. Recently, a large number of biodegradable PUs were developed using biodegradable polyols, such as poly(lactic acid) (PLA), polycaprolactone (PCL), poly(glycolic acid) (PGA), poly(hydroxyalkanoates) (PHA) to create new functional materials for biomedical applications.

The low molar mass chain extenders have a significant impact on the overall physical and mechanical properties of polyurethanes. Polyurethanes with no chain extension have no microphase separation between the hard and soft segments and consequently produce materials with very poor mechanical properties and thermal stability. The chain extender is commonly used to increase the length of both the hard and soft segments to increase the molar mass and enhance microphase separation. Consequently, a considerable increase in viscosity, modulus and glass transition temperature (*T_g_*) is obtained in the produced polyurethanes. Only polyurethane thermosets synthesized from polyols with more than two hydroxyl groups, such as plant oil polyols, do not require chain extenders due to the huge molar mass and good thermomechanical and viscoelastic properties of the obtained thermosets polyurethane elastomers. The chain extenders are low molar mass aliphatic and aromatic diols and diamines. Soft polyurethanes are commonly synthesized with aliphatic chain extenders compared to those prepared from their aromatic counterparts [47]. Chain extenders can be categorized into two classes: aromatic diol and diamine, and the corresponding aliphatic diol and diamine. In general, polyurethanes that are chain-extended with an aliphatic diol or diamine, produce softer materials than their aromatic chain-extended counterparts [47]. In the next section, the antimicrobial biodegradable PUDs and their thin films obtained from the solution cast will be summarized. The effect of different internal surfactants of amino polyols on the dispersion morphology and antimicrobial behavior will be demonstrated for selected systems. In addition, the influence of the different acids as neutralizing agents on the performance of bio-based cationic PUDs will be presented.

## 2. PUDs with Antimicrobial Behavior

Structural modifications of polyurethanes are required to improve the dispersibility of the hydrophobic chains of polyurethanes in water [16,48]. Poor dispersion stability of polyurethanes in water was observed with the aid of a protective colloid and an external emulsifier, even under strong shear force in attempt to disperse the polymer. It is well known that polyurethane with chemically bonded internal surfactant can be used to efficiently produce nanoscale polyurethane particles with excellent dispersion stability and homogenous distribution in water. The internal surfactants or emulsifiers are hydrophilic low molar mass diols with different ionic groups, such as quaternary ammonium salt, sulfonate, or carboxylate compounds. Some of them have non-ionic groups, such as poly(ethylene oxide). For anionic PUDs, a DMPA is one of the most common internal emulsifiers that can be reacted with diisocyanate and polyols to produce prepolymer. After that, a triethylamine (TEA) is typically used to neutralize the carboxylic acid groups to form the ionic function groups required for the production of PUDs that are stable in water [49,50,51,52,53,54,55,56,57,58,59,60]. 

Cationic PUDs are another class of aqueous PUDs, synthesized by replacing the low molar mass carboxylic acid diol emulsifier with a tertiary amine diol, followed by neutralization with an acid. These cationic PUDs have unique antimicrobial behaviors and exhibit excellent adhesion properties [61,62,63]. The cationic antimicrobial materials are able to bind to microbes or bacteria and disrupt cell structure, resulting in permeabilization and death [61,62,63]. Both anionic and cationic PUDs exhibit several advantages to polyurethane molecules: (i) the hydrophilic modified polyurethane can be dispersed under mild agitation speed; (ii) nanoscale particle size; (iii) excellent dispersion stability; (iv) the solid films obtained from the PUDs have improved solvent and water resistance [64,65,66]. 

Antimicrobial polyurethanes have wide ranging industrial applications, such as self-sanitizing medical devices, environmental surfaces, food packaging, etc. [67]. In these applications, purified and cleaned surfaces for the killing of pathogens or prevention of surface colonization are very essential. In general, materials containing ammonium salts and quaternary phosphonium exhibit strong antimicrobial behavior. In addition, polyurethane with coordinated silver was reported to greatly inhibit the growth of Staphylococcus epidermidis and Pseudomonas aeruginosa for at least one month [68]. Furthermore, polymer materials containing copper, zinc, or iron cations also showed satisfactory antimicrobial properties [69].

**Figure 3:**
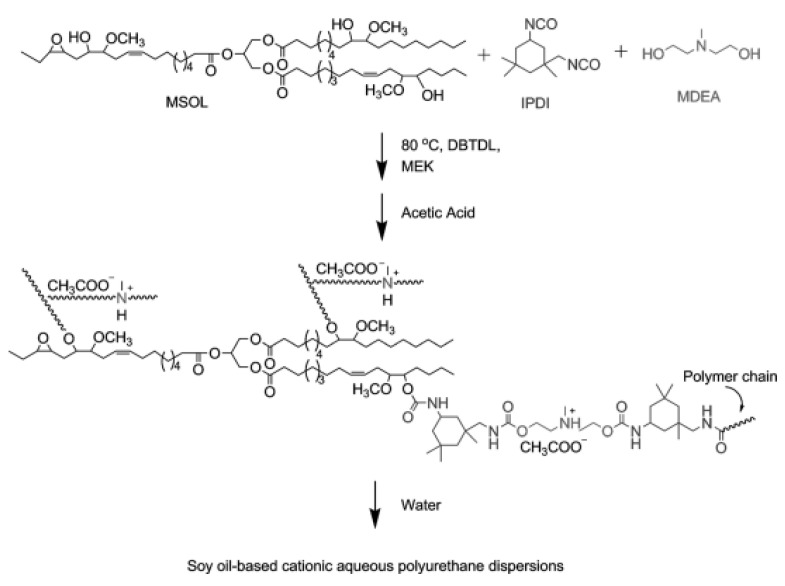
Reaction scheme of the cationic plant-oil based polyurethanes (reproduced with permission from [70]).

Antibacterial soybean oil-based PUDs and thin films were synthesized with different molar rations of an amine diol internal emulsifier [70]. Both PUDs and their thin films exhibited inhibitory activity against a number of foodborne pathogens. The authors observed that the antimicrobial behavior improved with increasing the ratio of ammonium cations. In addition, by decreasing the hydroxyl number of soybean-oil, the obtained polyurethane films become softer and the crosslink density considerably decreased with an enhancement in antimicrobial behavior. Although, on the other hand, the mechanical properties and *T_g_* of the polyurethane-thin films increased with increasing the molar ratios of the amine diol and the diisocyanate [70]. The main goal of this study was to synthesize soybean oil-based PUDs and thin films with antibacterial behavior as potential coatings for surfaces in food processing or healthcare environments, or for use as elements in food packaging for pathogen control. The PUDs were synthesized from the commercially available methoxylated soybean polyols (MSOL), isophorone diisocyanate (IPDI), and *N*-methyldiethanolamine (MDEA, internal surfactant). Figure 3 shows the reaction scheme of the cationic polymerization of antimicrobial soybean-based PUDs. 

**Figure 4:**
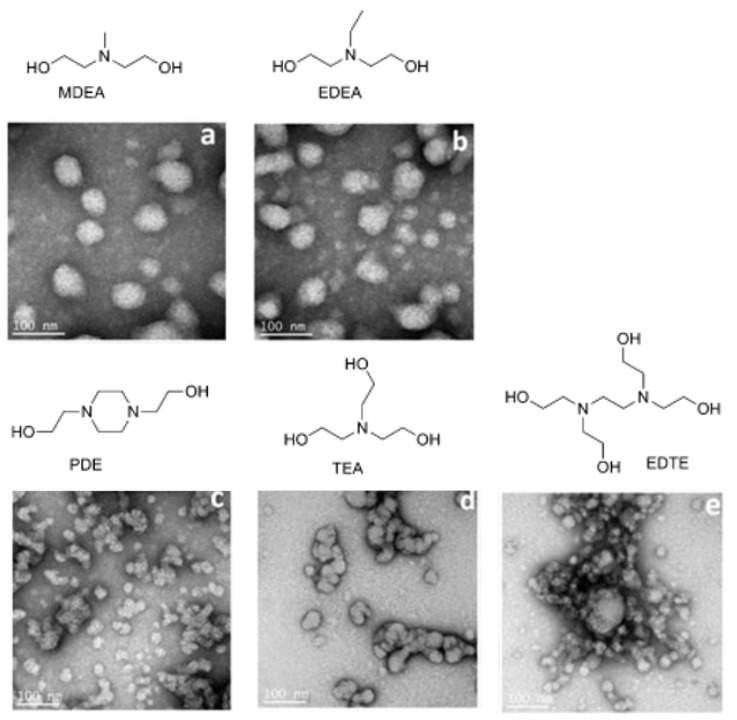
Structure of amino polyols used in the preparation of the polyurethane dispersions (PUDs) and their corresponding polyurethanes negative stained TEM images (all scale bars are 100 nm). The **a**, **b**, **c**, **d**, and **e** are related to the chemical structures of the amino diols on the top of each TEM image (adapted with permission from [71]).

Five different amino polyols, namely MDEA, N-ethyldiethanolamine (EDEA), 1,4-piperazinediethanol (PDE), triethanolamine (TEA), and 2,2′,2″,2′″(ethylenedinitrilo)tetraethanol (EDTE) have been used to successfully synthesize waterborne, antibacterial soybean oil-based cationic polyurethane coatings [71]. The different structures of amino polyols were found to have a significant impact on the morphology of the nanoparticle, mechanical properties, thermal stability, and antibacterial properties of the resulting coatings. The PUDs and their thin films obtained from the different amino polyols showed good antibacterial properties towards a panel of bacterial pathogens comprised of Listeria monocytogenes NADC 2045, Salmonella typhimurium ATCC 13311, and Salmonella minnesota R613. S. The five different chemical structures of the amino polyols and their polyurethanes negative-stained TEM images are shown in Figure 4. The morphology and particle size are strongly dependent on the nature of the amino polyols. Clearly the PUDSs with MDEA and EDEA polyols have relatively uniform particle size distributions with an average particle size of approximately 60 nm. However, on the other hand, the PUDs with PDE amino polyol have an average particle size of approximately 20 nm. This experimental finding was attributed to the fact that the PUDs with PDE have twice as many water-soluble ammonium ions as those prepared from MDEA and EDEA. Particle aggregation was observed for PUDs prepared with MDEA and EDTE amino polyols due to the higher hydroxyl functionality and higher cross-link densities relative to PUDs prepared from the other three amino polyols. Relative to the other four amino polyols, the polyurethane-MDEA-TEA dispersion has the lowest amount of ammonium cations, which also contributes to its large particle size and particles aggregation [71].

The nature of the neutralizing agent for the internal surfactant (amino polyols) might have a strong influence on the overlap properties and performance of the final products. Very recently, the effects of different acids as neutralizing agents in tailoring the performance of bio-based cationic PUDs have been reported by Zhang et al. [72]. Polyurethane films ranging from flexible elastomers to tough plastics were obtained, and their properties were controlled by the selection of the different acids. The PUDs were synthesized from castor oil polyols, IPDI, MDEA (internal surfactant), and different neutralizing agents including acetic, glycolic, hydrochloric, aspartic, and glutamic acids [72]. The tunability of the hydrogen bonding strength confers the resulting polyurethane films enhanced multi-functional properties, such as UV absorption, anticorrosion, and long-term antibacterial performance. The effect of polyols and the ionic chain extender contents on the antibacterial properties of the polyurethane films was investigated. The authors found that, all the polyurethane films exhibited enhanced antibacterial activity against *Vibrio parahaemolyticus* with an increase in MDEA content and a reduction in polyol functionality [72].

A novel UV-curable cationic waterborne polyurethane with pendant amine from 4-NCO prepolymer and modified by guanidino acetic acid (GAA) was synthesized by Du et al. [73]. The prepolymer originated from the progressively grafting of tridentate PCL. The different concentrations of GAA (0, 0.25, 0.5, 0.75, and 1.0 wt.%) were found to have an important and positive reinforced role on the self-antibacterial coatings [73]. The obtained PUDs showed excellent properties in Gram-negative (92.05%) and Gram-positive (94.77%) antibacterial tests. Compared with the linear amine waterborne polyurethane, PUDs have significant superiority in terms of stability, and the increase in antibacterial efficiency is within the region of 50%. In addition, the antibacterial efficiency was about 87.94% after washing 12 times. The atomic force microscopy (AFM) results showed that the GAA and pendant amine enhanced the antibacterial performance.

**Figure 5:**
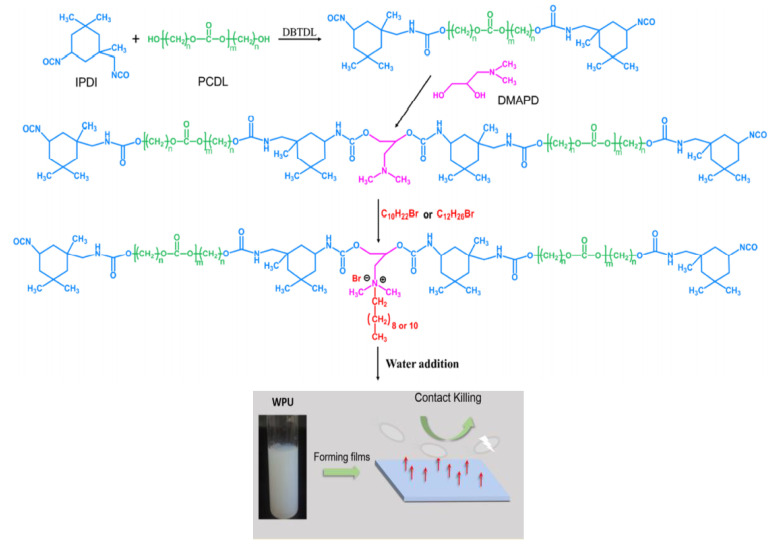
Elementary steps for the synthesis of waterborne cationic polycarbonate-based PUDs. Modified from (adapted with permission from [74]).

Polycarbonate diol (PCDL), IPDI, and 3-dimethylamino-1,2-propanediol (DMAPD) as the internal surfactant were used to synthesize cationic waterborne PUDs. DMAPD was reacted with 1-bromodecane and 1-bromododecane, to produce a quaternary ammonium salt and this was dispersed in water to produce cationic PUDs with nanoparticle size, high dispersion stability, and excellent antimicrobial properties [74]. Figure 5 shows the elementary steps of the synthesize process of waterborne cationic PC-based PUDs. The casted solid films showed a high efficiency in killing Gram-negative *Escherichia coli* and Gram-positive *Staphylococcus epidermidis* [74]. The longer hydrocarbon tail of this polyurethane structure provided higher antimicrobial potency due to the strong hydrophobic interactions. In addition, the authors found that the antimicrobial action of the solid films was based on contact killing, without leaching of bactericidal species, as revealed by a zone-of inhibition test [74]. Furthermore, the polyurethane films obtained from the solvent cast exhibited good mechanical properties with a tensile strength of 36 MPa and an elongation at break of 620%. The excellent antimicrobial and mechanical properties of the solid films of PC-based polyurethane might be suitable for antimicrobial coating applications [74]. The biodegradation behavior of solid PU films obtained from the solution cast of the different aqueous PUDs will be reviewed in the next section.

## 3. Biodegradable PUDs and Their Solid Films 

Aqueous biodegradable PUDs and their cast films with tunable mechanical and physicochemical properties have been widely used in the rapidly growing market of resorbable materials. The degradation behavior of PUDs and their films are strongly dependent on the chemical structure of the PU backbone, particularly the type of polyols or the soft segments. Generally, structure modification of PU, such as incorporation of hydrolytically or enzymatically cleavable groups into the PU structure confers biodegradability [75]. The PUDs synthesized from soft segments, such as poly(lactic acid) (PLA), polycaprolactone (PCL), poly(glycolic acid) (PGA), polyethylene glycol (PEG), poly(butylene succinate-*co*-adipate) (PBSA), poly(hydroxyalkanoates) (PHA), poly(hydroxybutyrate) (PHB), and their copolymers are biodegradable. For the successful chemical modification of polyurethane, to improve its biodegradability, it is crucial to maintain a good balance between the desire mechanical properties and biodegradation rate [75]. Biodegradable polyurethanes have been widely used in many biomedical applications, such as scaffold materials for bone repair [76], cartilage [77,78], and blood vessels [79]. In addition, the polyurethanes can be manufactured via 3D printing techniques and the chemical structure can be tailored for excellent biocompatibility, good flexibility as well as excellent mechanical properties and biodegradability [80,81,82,83,84]. In addition, the PUDs can be synthesized with certain soft and hard segments, enabling the shape-memory capability of the solid films.

**Figure 6:**
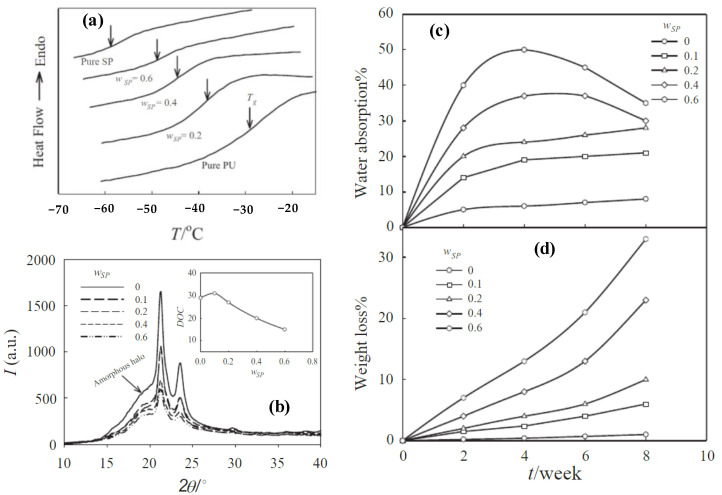
(**a**) DSC thermograms of second heating run for PU/soy protein (SP) blends of different compositions. The measurements were carried out at 10 °C·min^−1^ heating rate. The arrows denote the *T_gs_* for each composition. (**b**) WAXS patterns for PU/SP blends of different concentrations. Inset-plot shows the composition dependence of degree of crystallinity (DOC) for PU/SP blends calculated from the analysis of WAXS patterns. (**c**) Water absorption and weight loss percentages (**d**) of PU/SP blends as a function of degradation time in buffer solution of pH = 7.4 at 37 °C (adapted with permission from [85]).

Waterborne PCL-based PUDs and soy protein (SP) were homogenously mixed in an aqueous solution to prepare hydrolytically degradable binary miscible PU/SP blends. The PU and SP were found to have miscible mixtures over the entire range of composition. As clearly seen in Figure 6a, the DSC (differential scanning calorimeter) data showed only one *T_g_* that was shifted to a lower temperature with increasing SP content in the blend. The degree of crystallinity measured by the WAXS (wide angle x-ray scattering) increased to reach a maximum value of 10 wt.% SP and then decreased linearly with increasing the concentration of SP, as demonstrated in Figure 6b [85]. The significant decrease in the degree of crystallinity (DOC) observed in many binary miscible blends was not observed for the current system (PU/SP blend) due to the presence of nano-aggregates of the processing aids, which might act as nucleating agents. The results for hydrolytic degradation in a buffer solution (pH = 7.4 at 37 °C) for all samples are shown in Figure 6c,d as the degradation time dependence on water absorption% and weight loss%, respectively. The water absorption increased significantly with increasing SP content at a constant degradation time, as clearly seen in Figure 6c. It is also clear that the water absorption of the blend with 20 wt.% SP increased substantially during the first week of the test and then increased slightly. For blends with concentrations ≥ 40 wt.% SP, the water absorption reached a maximum after approximately 4 weeks and then decreased significantly with increasing degradation time. The relative weight loss increased significantly with increasing degradation time and SP content in the blends as seen in Figure 6d. The hydrolytic degradation of polyester urethane is known to be due to the degradation of the polyester segments (PCL soft segments).

**Figure 7:**
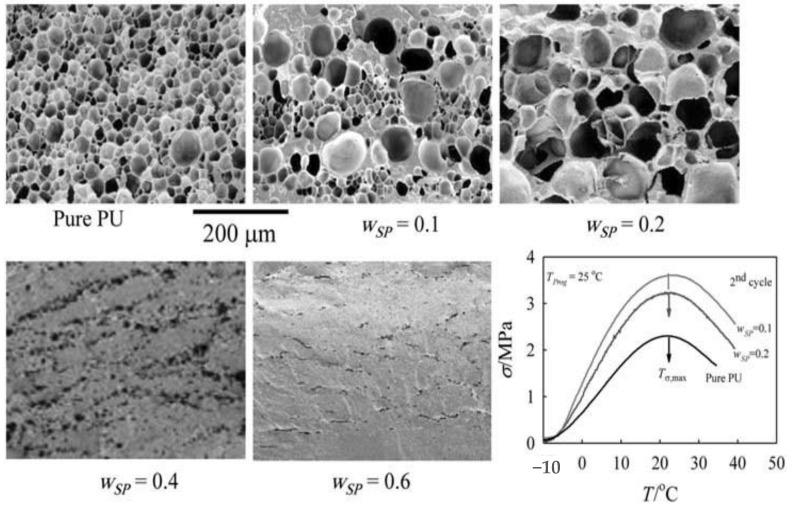
SEM micrographs for the porous structure of PU/SP blends of different concentrations that were generated using scCO_2_ technique at 35 °C foaming temperature, 100 bar, and 30 min saturation time. Temperature dependence of stress for the thermomechanical cycle under constant strain condition for PU/SP blends of different concentrations. The measurements were carried out at *T*_prog_ = 25 °C, *ε*_m_ = 100%, *T*_low_ = −10 °C, and *T*_high_ = 50 °C. The arrows denoted the values of *T_σ,_*_max_ for each composition (reproduced with permission from [85]).

For the shape-memory effect of the PU/SP blends, the values of the shape fixity rate (*R*_f_) and switching temperature (*T*_sw_) for different blend compositions obtained from the thermomechanical cycles under stress-free condition were found to be SP independent. However, on the other hand the shape recovery rate (*R*_r_) decreased from 80% for pure PU, up to 67% SP wt.%. This decrease in the value of *R*_r_ was attributed to the decreasing hard domain content contributed by the PU component. Under constant strain conditions, the maximum stress recovery (*σ*_max_) increased with increasing the content of SP, reaching a maximum at 10 wt.% SP (see Figure 7). This experimental fact was in good agreement with the maximum DOC determined by WAXS analysis. 

Porous structure scaffolds of PU/SP blends with different composition were fabricated by the high-pressure supercritical carbon dioxide (scCO_2_) foaming technique. The degree of porosity reached approximately ≈ 80 ± 5% for pure PU and decreased with increasing SP content, where no porous structure could be obtained at 40 wt.% SP due to the decreasing stability of the foams under the applied processing conditions, as seen in Figure 7 [85]. The polymer films and foams obtained from this hydrolytically degradable binary miscible blend of PU and SP with improved crystallization behavior as well as hydrolytic degradability and shape-memory capability can be considered as an interesting multifunctional candidate material for potential biomedical applications [85].

**Figure 8:**
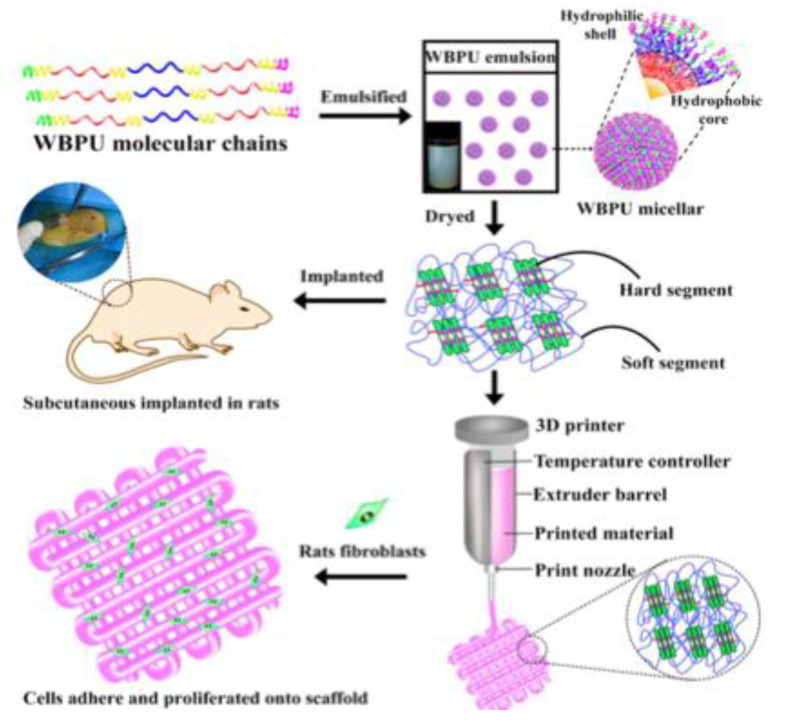
The self-assembled micellar structure of WBPU (PUDs) and the bioprinting process of WBPU by a self-developed platform (reproduced with permission from [86]).

Degradable PUDs with modified amino acids were successfully employed for making scaffold using deposition 3D printing technology at a low temperature range of 50~70 °C for biomedical applications [86]. The PUDs of this study were synthesized with different degradation behaviors based on the content of the hydrophilic chain extenders. The PUDs were synthesized from degradable poly(butylene glycol adipate)diol (PBGA), polyethylene glycol (PEG), and IPDI using the self-emulsifying technique [86]. Figure 8 shows a schematic diagram for the process of making PUDs with micellar structure. The figure also shows that the polyurethane structure is a segmented multiblock of soft and hard segments that can be used directly for biomedical applications. In addition, the authors found that the polyurethane scaffolds are highly applicable for implantable tissue engineering due to the biocompatibility of the degradable fragments of the polyurethane. The degradability of PU scaffolds was investigated based on measuring the mass loss, the pH value of the degradation solutions, the change in chemical structure, and the crystallinity during the degradation process. The degradation solutions were also collected to evaluate their effects on cells. The PUDs were investigated using FTIR spectroscopy, H^1^ NMR spectroscopy, atomic force microscopy (AFM), particle size, and zeta electric potential analysis. In addition, the effect of a hydrophilic chain extender (DMPA) on the thermal properties, water absorption, surface, and mechanical properties was systematically investigated [86]. 

**Figure 9:**
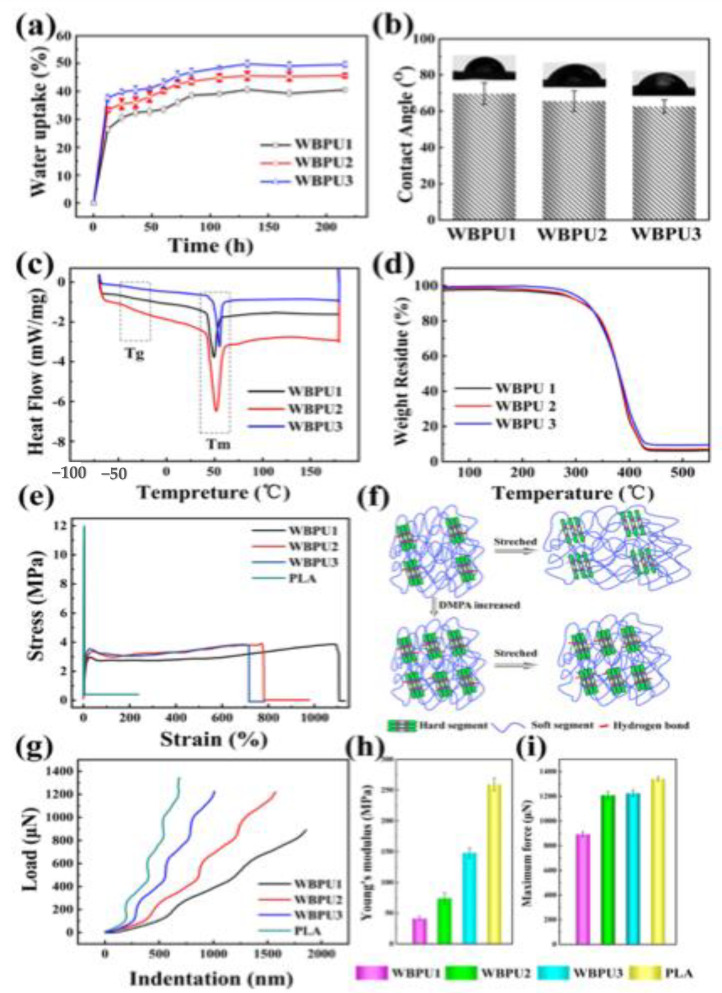
(**a**) The water uptake. (**b**) Water contact angle. (**c**) Thermal stability (TGA) and (**d**) DSC thermograms of WBPU films with different dimethylol propionic acid (DMPA) content. (**e**) The stress–strain curves of WBPU and PLA films. (**f**) The schematic diagram of the molecular chain structure during tensile mechanics. (**g**) The load-indentation curves of WBPU and PLA films. (**h**) The Young’s modulus of WBPU and PLA films. (**i**) The maximum force of WBPU and PLA films (reproduced with permission from [86]).

The water absorption properties in distilled water of the thin films obtained from the solvent cast of the PUDs have been investigated. The water absorption test is commonly used to measure hydrolytic degradation. In this case, water absorption was evaluated for polyurethane-thin films of different DMPA contents [86]. The measurements were carried out for up to 250 h for three samples of different DMPC contents. WBPU1, WBPU2 and WBPU3 represent the solid films obtained from the waterborne PUDs with 4, 5 and 6 wt.% DMPA, respectively. The water absorption increased dramatically after about 25 h for all three samples, while the magnitude of water absorption was more significant for the film with a high content of DPMA, see Figure 9a. With the increase in DMPA content, the water absorption rate increased due to increases in the hydrophilic groups (DMPA) in the polyurethane chains, and the films being more hydrophilic [86]. The water contact angle was also found to be DMPC content dependent (i.e., the water contact angle decreased with increasing the content of DMPC as clearly seen in Figure 9b). Both melting peaks and *T_gs_* (DSC) and thermal stability (TGA) showed insignificant changes with increasing DMPA, as seen in Figure 9c,d, respectively [86]. 

The stress–strain curves for PLA and polyurethane films obtained from the solvent cast are shown in Figure 9e. Clearly, the PLA film is much stiffer and has a significantly higher stress at break and a lower elongation at break than the polyurethane films. This is an expected behavior due to the elastic nature of polyurethane films in comparison with the stiff PLA. It is also clear that the tensile strength of polyurethane films increased significantly with increasing the DMPA content. Although, on the other hand, the elongation at break decreased with the increase in DMPA content. This experimental fact was attributed to the increase in the hard segment content with increasing the concentration of DMPA. In addition, the Coulomb force and hydrogen bonding between the molecular chains were enhanced with the increase in DMPA, as described schematically in the related mechanism in Figure 9f. The effect of DMPC on the load-indentation, Young’s modulus, and maximum force is shown in Figure 9g–i, respectively. The figures also show the data for PLA. The Young’s modulus and maximum force increased significantly with increasing DMPA and reached a maximum for PLA. Due to the high modulus and hardness of PLA, its degraded small pieces may possibly scratch the surrounding tissues in vivo. The polyurethane films have lower hardness and higher elasticity, which means it may more easily tightly comply with the surrounding tissue, and the degraded pieces will not damage the surrounding tissue [86]. 

**Figure 10:**
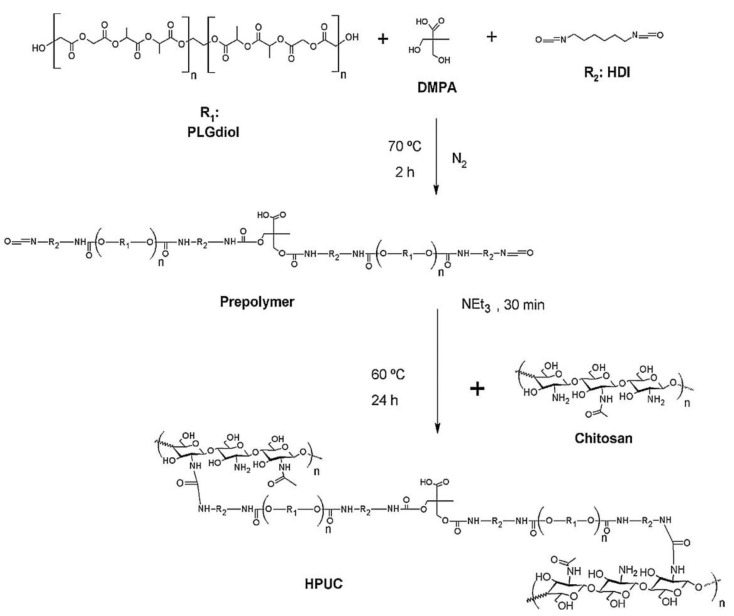
Elementary steps of synthesis of chitosan-polyurethane hydrogel membrane (HPUC) (reproduced with permission from [87]).

Waterborne dispersions of a chitosan-polyurethane hydrogel membrane (HPUC) with low cytotoxicity and improved wound healing abilities when used with mononuclear bone marrow fraction cells in a diabetic rat model, has been developed by Viezzer et al. [87]. The HPUC was constructed as a combination of chitosan blocks and biodegradable polyurethane. The authors claimed that the HPUCs have interesting properties that make them suitable for wound healing applications. The polyurethane prepolymers were synthesized from hexamethylene diisocyanate (HDI), DMPA, poly(lactide-*co*-glycolide) diol (PLGA diol), and triethylamine (TEA). Chitosan (0.01 g/mL) solution in 1.0% acetic was added dropwise to the polyurethane prepolymer solution under stirring at 70 °C to synthesize the HPUC. Figure 10 shows the elementary steps for the synthesis of HPUC. The HPUC1, HPUC2, and HPUC3 samples have 0.25, 0.5, and 0.75 g of PLGA-diol, respectively.

**Figure 11:**
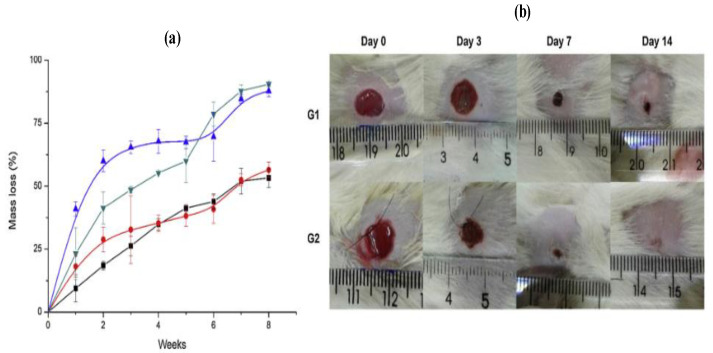
(**a**) pure chitosan film (CHS) and HPUC mass loss curves after up to eight weeks: ◼ CHS, ● HPUC1, ▼HPUC2 and ▲HPUC3. All values are expressed as mean ± standard deviation (n = 3). (**b**) Representative image in the effects of HPUC1 and mononuclear bone marrow fraction isolation (BMMNCs) on wound healing in diabetic ulcer model. (A) Macroscopic observations in wound areas in the control (G1) and wound healing contraction induced by HPUC1 plus BMMNCs (G2) (adapted with permission from [87]).

The degradation behavior of HPUC in phosphate buffered saline, PBS (pH 7.4) for up to eight weeks is shown in Figure 11a. The membrane weight loss has two different mechanisms. Since the membranes were not neutralized at the end of the synthesis, the chitosan chain amine groups are protonated, and consequently, the chitosan would be slowly dissolved in the aqueous solution. Therefore, the pure chitosan film (CHS) presents a continuous weight loss of up to 53.2%, which cannot be explained by hydrolysis [87]. The authors also found that both HPUC2 and HPUC3 membranes totally degraded during the eight weeks, as seen clearly in Figure 11a.

The viability of using HPUC for biomedical applications, such as tissue engineering has been investigated [87]. Two groups of diabetic animals with wounds have been used in this study [87]. The first group (G1) consisted of diabetic animals with a wound without treatment, while the second group (G2) consisted of diabetic animals with HPUC and BMMNCs (mononuclear bone marrow fraction isolation) transplanted onto the wound site. Significantly better healing was observed after 7 days for the group of animals treated with the HPUC combined with the injection of BMMNCs in the interphase between the tissue and the HPUC membrane (G2) compared to the untreated one (G1), as clearly seen in Figure 11b. After 14 days, a clean and dry wound with a better regeneration rate and a significant reduction in wound size was observed macroscopically in G2 compared to G1 (see Figure 11b). These data suggest that the HPUC membrane achieved hemostasis of the wound, supporting the formation of the fibrin matrix and cell infiltration [88]. BMMNCs are commonly used to enhance tissue regeneration and reduce wound size in rat diabetes ulcer models of streptozotocin. The 14 days required for complete wound healing in G2 is significantly less time or a significantly faster healing process compared to what was previously published in the literature [89]. 

Biodegradable waterborne PUDs and their films were synthesized from PCL-diol (80%) and poly(hydroxybutyrate) (PHB) diols (20%) soft segments [90]. Three different molar masses of PHB-diol, namely, 1352, 1679, and 2062 g/mol were used in this study. It has been observed that the PCL-PHB-based polyurethane films obtained from the solvent cast have 3–7% crystallinity, good flexibility and shape-memory capability. The polyurethane films also showed excellent biodegradation of 52% weight loss after 30 days and biocompatibility (capsule thickness B23 mm) in vivo. The dispersions were also used successfully to prepare nanofibers using the electrospinning technique, without the use of organic solvents [90]. These eco-friendly polyurethane containing PHB and PCL blocks have good mechanical properties, processing abilities, and biocompatibility, and might be used as a new category of biodegradable elastomers and potential biomaterials for cardiovascular and other medical applications [90].

Waterborne polyether-based PUDs (PEUR) grafted with modified gelatin hydrolysate (GH) have been developed as scaffolds for biomedical applications [91]. The modified PU-grafted GH showed good toughness and hydrophobicity, as well as good thermal stability, excellent biocompatibility, and good structural homogeneity. The degradation rate in a buffer solution was found to be GH dependent (i.e., the rate of hydrolytic degradation increases significantly with increasing the concentration of GH). The modified PEUR-g-GH was completely degraded in the buffer solution after 60–90 days, which could meet degradation requirement for tissue engineering scaffold materials [91]. 

**Figure 12:**
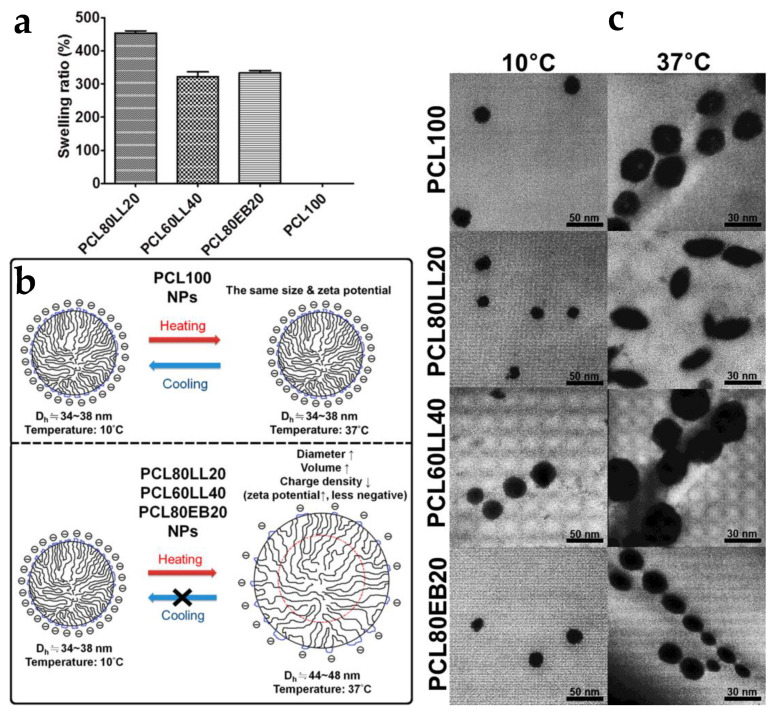
(**a**) Equilibrium swelling ratio (%) of waterborne biodegradable PU NP dispersions at 37 °C. (**b**) A summary of swelling and the size change for the PU NPs. (**c**) TEM images for various PU NPs at two different temperatures, 10 and 37 °C (adapted with permission from [92]).

Waterborne PUDs based on different biodegradable oligo-diols as soft segments were synthesized as nanoparticles (NPs) [92]. Thermally induced swelling and self-assembly of these NPs were observed and associated with hydrogen bonding and the degree of crystallinity. The thermo-responsiveness of the polyurethane NPs with mixed biodegradable oligo-diols may be employed to design smart biodegradable carriers for delivery of cells or drugs near body temperature [92]. The PUDs were made from PCL diol, and polyethylene butylene adipate diol (PEBA diol) or PLLA diols. The molecular ratio of the two oligo-diols, of the soft segments was found to have a significant impact on the polyurethane NPs. The highest swelling ratio (~450%) was observed for PCL80/PLLA20 dispersion (Figure 12a). The temperature-induced swelling phenomenon was consistent with the size and volumetric changes. A summary of size alterations for different polyurethane NPs is presented in Figure 12b. The PCL100 NPs had no thermal responsiveness, whereas the other three polyurethane NPs showed an increase in particle size and zeta potential upon heating. The effect of soft segments on the particle size of polyurethane in aqueous dispersion was investigated using TEM, as seen in Figure 12c. 

Scaffolds for tissue engineering with sequential drug release functionality were fabricated from water-based 3D printing ink containing biodegradable polyurethane, chemokine SDF- 1, and Y27632 drug-embedding polyurethane microspheres [93]. In this study, the PCL-diol and poly(ethylene adipate) glycol were used as soft segments and IPDI as a hard segment in the biodegradable aqueous PUDs. The scaffolds were synthesized with 200 ng/mL SDF-1 and 22 wt.% Y27632-encapsulated microspheres (55 μg/mL Y27632 in microspheres) had the optimal performance. The authors claimed that the 3D printed scaffolds, with sequential releases of SDF-1 and Y27632, may have potential applications in cartilage tissue engineering [93]. 

Biodegradable transparent PUDs were synthesized using different bio-derived precursors, such as PEG-600, dimer acid, castor oil, and glycerol. Three different compositions of PUDs were prepared by varying the amount of 1, 4-butanediol (BD), IPDI, and glycerol-dimer acid modified citric acid-based polyol. Different techniques including FTIR, XRD, ^1^H NMR, and ^13^C NMR were conducted to sanguine the successful synthesis of the PUDs. Moreover, the specific gravity, mechanical property measurements as well as gel permission chromatography (GPC), thermogravimetric analysis (TGA), and DSC studies were performed to obtain detailed information about the physical, mechanical, and thermal properties of the as-synthesized PUDs [94].

A series of waterborne biodegradable PCL-based PUDs with different ratios of ionic groups (i.e., different concentrations of DMPA as internal surfactant) were synthesized [95]. All cast films from PUDs were found to be non-cytotoxic in the cell viability test and had suitable physicochemical and mechanical properties based on the measurement of zeta potential, water contact angle, mechanical properties, water absorption, thickness change, and gelatin test [95]. The initial molar masses of PCL and polyurethane obtained from GPC analysis were about 120,000 and 600,000 g/mol, respectively. The molar mass of PCL after the four-week degradation time remained almost unchanged, but that of polyurethane decreased to about 80%. The degradation of PCL and polyurethane showed statistically significant differences [95]. 

Biodegradable PUDs were synthesized based on castor oil polyols (CO), polyester polyols, PEG, and 1,6-hexamethylene diisocyanate [96]. The effects of different types of polyols on the biodegradation behavior and physicomechanical properties of PUDs and their films were investigated [96]. The biodegradability behavior was investigated by hydrolytic and enzymatic degradation in PBS and with lipase enzyme, respectively. The nature of polyols was found to be an important factor to control the polarity of the PU films and their biodegradation rates. Both castor oil-based polyurethane (CO and CO 1233) samples showed the lowest rate of degradation due to the lower polarity compared to the other polyols. For example, for PU with only a PEG soft segment, the degradation rate increased significantly. Polyurethanes with CO and PEG soft segments showed an increase in the degradation rate based on the concentration of PEG in the polyol mixtures. The degradation rate of samples was related to the water absorption ability, with more degradation found in PBS. Different behaviors have been observed for the enzymatic degradation of PU films. The PU with a mixture of castor oil and PEG showed the highest degradation rate, while the polyurethane with PEG only showed the lowest enzymatic degradation rate. This experimental fact was attributed to the different degradation mechanisms of polyurethane films in PBS and in lipase environment (enzymatic). The enzymatic degradation process is commonly carried out via hydrolysis, which should be very significant for the ester bonds in triacylglycerol in castor oil compared to the PEG. The castor oil and CO 1233 sample, which contained polyester polyol and castor oil in its structure, showed better degradation in the lipase environment than in PBS [96]. Lipase catalyzes the hydrolysis of ester bonds in triacylglycerol effectively and did not show any effect on hydrolysis of PEG sample with the relatively stable etheric functional group in polyethylene glycol. 

**Figure 13:**
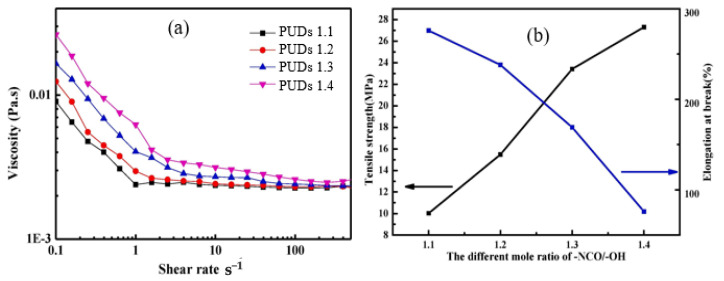
(**a**) shear rate dependence of PUDs viscosity at 25 °C for different R values. (**b**) Effect of R value on the tensile strength and elongation at break of different PU films (adapted with permission from [97]).

Highly degradable soybean oil-based waterborne with excellent mechanical properties, thermal stability was recently synthesized by Yang et al. [97]. The degree of hydrophilicity of casted films was adjusted by changing the R value (the molar ratio of –NCO/–OH). The PUDs were fabricated with no solvent and catalyst using epoxidized soybean oil, ricinoleic acid, IPDI, DMPA (internal surfactant), TEA, and ethylenediamine (EDA). The authors found that the tensile strength of the cast solid films significantly increased from 10.02 to 27.32 MPa with increasing the R value. Similar increases in the *T_g_* and thermal stability of the casted films were observed with increasing the R value. The degradation behavior of the casted films was investigated in an aqueous solution of 3.0 wt.% sodium hydroxide solution at 45 ℃. The effect of R value on the viscosity of the PUDs is shown in Figure 13a. Clearly, both the R value and shear rate have a strong influence on the rheological behavior of the PUDs. The viscosity considerably decreased with increasing shear rate, regardless the different values of R. The magnitude of decreasing viscosity reduced in the high shear rate range. With increasing the R value, the viscosity of the dispersion increased, particularly at the low shear rate range. The effect of shear rate and R on the viscosity of the dispersion is almost eliminated at high very high shear rate (higher than 100 s^−1^). When the R values increased, the cross-linking degree of polyurethane was enhanced, strengthening the interaction of the polymer chain. In addition, with increases in the shear rate, the physical entanglements of the molecular chain gradually open, and the friction resistance between the polymer chains reduces, resulting in reduced viscosity [97].

The R value was found to have a strong influence on the mechanical properties of the casted films. The tensile strength increased linearly with increasing the R value, while the elongation at break decreased strongly with increasing the R value (see Figure 13b). The obtained data were attributed to the fact that, as the R value increased, the degree of crosslink density of the polyurethane film will be increased, and the interaction of the molecular chains will become stronger. Thereby, the tensile strength of the polyurethane film was improved and the elongation at break decreased. 

**Figure 14:**
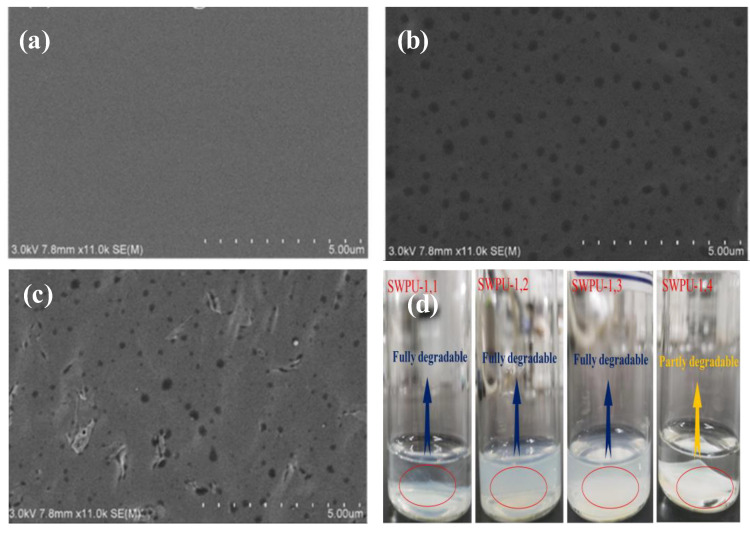
SEM images of PUDs films: (**a**) Before degradation; (**b**) PUDs-1.2 film after 24 h degradation time; (**c**) PUDs-1.4 film after 24 h degradation time; (**d**) PUDs films in a 5 wt.% NaOH solution for 8 h (adapted with permission from [97]).

The SEM photograph of polyurethane film before the degradation process is shown in Figure 14a. The SEM shows a very smooth surface with no morphology before the degradation process. A porous structure was observed for the polyurethane films after the degradation process. For example, the SEM photographs for polyurethane films with 1.2 and 1.4 R values after hydrolytically degradation in NaOH solution (3.0 wt.%) at 45 °C for 24 h showed porous structures, as clearly seen in Figure 14b,c, respectively. The porous structure is an indication for the hydrolytic degradation which was accelerated by the reaction of NaOH with the ester groups of the PU (saponification process). The degradation process of polyurethane films of different R values was also investigated in concentrated NaOH (5 wt.%), as seen in Figure 14 d. The authors found that all films with 1.1, 1.2, and 1.3 R values were fully degraded after 8 h. Only polyurethane film with a 1.4 R value was partially degraded due to its high crosslink density [97].

Biodegradable waterborne PUDs based-on different molar masses of PEG and PCL were recently developed to increase the solid content in the dispersions [98]. The biodegradability of the solid films was only observed in a mixture of PBS and lipase and no degradation was found in PBS only. Different association relationships between the DMPA and a nonionic hydrophilic PEG were found to have strong influence on the solid content of the dispersion and on the thermomechanical properties of the solid films. The association of PEG molecules with DMPA increased the crystallization behavior, tensile properties, and water and soil repellency of the polyurethane films. The solid content of the dispersion increased from 41% to 52.7%. In addition, PEG with molar masses of 400 and 1000 g/mol had the best effect on the dispersibility and stability of PUDs [98].

Amphiphilic biodegradable waterborne PUDs were synthesized by Yang et al. without using any organic solvents, catalysts or cross-linkers [99]. The hydrophobic PCL diol was replaced with different block lengths of hydrophilic PEG in the synthesis of PUDs. The chemical structure of the polyurethane films with ester and ether groups has amphiphilic characteristics, which is important for the regeneration and maintenance of tissue. The phase separation between soft and hard segments, mechanical and thermal properties, as well as the water absorption, biodegradability and cytotoxicity of polyurethane films were investigated as functions of the composition and content of the soft segments. It was found that by tuning the block length of PEG and content of soft segments, the desired properties of polyurethane films could be achieved. The authors claimed that the amphiphilic and biodegradable polyurethanes described in this work could be very promising candidates for 3D printing materials for tissue engineering scaffolds [99].

## 4. Conclusions

In this review article, a comprehensive overview of recent developments and progress in PUDs and their films—obtained from solvent casts—in particular, degradation behavior, antimicrobial properties, and biomedical applications has been presented. Antimicrobial PUDs and their cast films can be considered as one of the most rapidly developing and active branches of PU chemistry and technology, as a result of their versatility and environmental friendliness. PUDs and films containing ammonium salts and quaternary phosphonium exhibited strong antimicrobial behavior. The antimicrobial and degradable PUDs and their films can be widely used in industrial applications, such as self-sanitizing medical devices, environmental surfaces, food packaging, etc. The degradation behavior of PUDs and their films are strongly dependent on the chemical structure of the polyurethane backbone, particularly the type of polyols or the soft segments and the polarity and concentration of the internal surfactant. Generally, structure modification of polyurethanes, such as incorporation of hydrolytically or enzymatically cleavable groups into the polyurethane structure confers biodegradability. For the successful chemical modification of polyurethane, it is crucial to improve the level of biodegradability to maintain a good balance between the desired mechanical properties and the biodegradation rate. The polyurethane scaffolds can be manufacturing via 3D printing techniques and the chemical structure can be tailored for excellent biocompatibility, good flexibility as well as excellent mechanical properties and biodegradability. Many examples of biodegradable PUDs and solid films with different soft segments, such as PEG, PLA, PLLA, PCL, PBGA, PLGA, PHB, PEBA, chitosan, gelatin, and castor oil have been summarized in this review article. Many of these examples showed biocompatibility and potential biomedical applications. Further developments in the chemistry and structural modifications of PUDs will continue to create new multifunctional, environmentally-friendly commercial products with outstanding properties, suitable for a wide range of industrial applications, which will have strong positive impacts on both sustainability and the polymer industry.

## Sample Availability

Not available.
